# Veterinary Medical Association of New Jersey

**Published:** 1887-01

**Authors:** Wm. Herbert Lowe

**Affiliations:** Secretary


### VETERINARY MEDICAL ASSOCIATION OF NEW JERSEY.

Veterinary surgeons, from almost every county in the State of New Jersey, met in convention at the American House, in the city of Trenton, on Thursday, the 9th day of December, 1886, to consider the subject of veterinary legislation.

The legislative committee, of which Dr. J. W. Hawk, of Newark, was chairman, met at 10 A.M. After considering the merits of several bills, the committee endorsed the bill drafted by Dr. Wm. Herbert Lowe, of Paterson. The object of the bill is to regulate the practice of veterinary medicine and surgery in the State of New Jersey, and is similar, in many respects, to the one in force in regard to practitioners of human medicine.

At 11.30 A.M. the regular session was called to order by President Miller, of Camden County. After calling the roll, Dr. Lowe read letters from Dr. W. H. Pendry, Dr. D. J. Dixon, and others, in which they expressed a deep interest in the affairs of the association.

The secretary read the minutes of the Long Branch meeting, which were adopted.

The treasurer, Dr. L. R. Sattler, of Newark, reported in regard to the finances of the association. The secretary made no report.

Dr. Miller next called for the report of the committee on veterinary legislation. The chairman stated that they were in favor of the bill drafted by Dr. Lowe. By this time, Dr. E. M. Hunt, secretary of the New Jersey State Board of Health, had arrived, and the president took advantage of the opportunity by requesting him to give his views upon the subject under consideration, before the association took action. Dr. Hunt said that the bill just recommended by the legislative committee was an excellent one, and that he approved of it in most respects. The third section of the bill is to the effect that any person who shall have been practicing veterinary medicine or surgery in the State, for a livelihood, for a period of not less than ten years immediately preceding the passage of the act, without having obtained a diploma from a legally chartered or incorporated veterinary college or university, as provided for in section 2 of the act, may, at the next regular meeting of the Veterinary Medical Association of New Jersey, present himself to the Board of Censors for examination, and if he should be found worthy, would be allowed to register and continue practice.

Dr. Hunt said that any person who had had twenty years' experience in the practice of *human* medicine or surgery in one locality in the State, immediately preceding the passage of a certain act of the Legislature, could make affidavit as to the facts and register and would be allowed to continue practice without undergoing *any* examination. He said that if the State Society of Veterinary Surgeons examined non-graduates, and gave them certificates testifying that they were worthy practitioners, that we would elevate their standing much more than we would by allowing them to register without any examination. If this latter course were adopted, he continued, it would be an easy matter to ascertain the professional standing of any particular practitioner, whether a graduate or not. The affidavits of non-graduates would be on file with the diplomas of graduates, and could be examined in the offices of the county clerks. Dr. Hunt's suggestions were approved of, and were incorporated in the bill when it was adopted by the association.

The Board of Censors reported in favor of R. E. Stanwood, of Freehold, and he was elected to membership. Several applications were made for membership, after which the following gentlemen were proposed for honorary membership: Rush Shippen Huidekoper, M.D., V.S., *Dean of the Veterinary Department of the University of Pennsylvania*, and Wm. L. Zuill, M.D., V.S., of the same institution, by Dr. Miller; Geh. Med. Prof. Dr. R. Leisering, Dresden, Saxony, by Dr. Sattler.

After the transaction of routine business the members of the association listened to an able address by Dr. Hunt, on veterinary matters in New Jersey. He told of the work being done by the State Board of Health in stamping out pleuro-pneumonia and other contagious animal diseases prevalent in the State. He spoke, at some length, of the benefits to be derived from the association—social, scientific and otherwise. He said that we, as veterinarians, were fortunate in not having any old troublesome laws to deal with, as had the practitioners of human medicine, and that, in his opinion, little legislation was needed beyond the passage of the bill which had been endorsed by the association. Dr. Hunt said that the medical men of the State had failed, except in getting a general law passed. He said that the association ought to be very careful who they admit to membership and consequently that a very important duty devolved upon the Board of Censors.

The Doctor extended an invitation to the members of the association to visit the library of the State Board of Health at any time. He further stated that a member would be allowed to take a veterinary work from the library upon the recommendation of the President or Secretary of the association.

The Chair appointed Drs. Hawk, Smith, Dustan, Cooper and Loblein, with Drs. Miller and Lowe, members ex-officio, as a committee on legislation, to represent the interests of the association at Trenton and to present the proposed bill to the Legislature.

Upon motion of Dr. Loblein, the secretary was instructed to have copies of the bill printed and sent to members of the association as well as to the Legislature.

The President appointed Dr. Loblein essayist for the next regular meeting.

The association decided to hold the April meeting in Newark. The place of meeting to be selected by a committee consisting of Drs. Sattler, Vogt and Hawk, all of Newark.

After a prolonged session the members adjourned for dinner.

WM. HERBERT LOWE, D.V.S., *Secretary.*

